# Identification of a multidimensional transcriptome signature for survival prediction of postoperative glioblastoma multiforme patients

**DOI:** 10.1186/s12967-018-1744-8

**Published:** 2018-12-20

**Authors:** Wei-Zhen Gao, Lie-Mei Guo, Tian-Qi Xu, Yu-Hua Yin, Feng Jia

**Affiliations:** Department of Neurosurgery, Renji Hospital, Shanghai Jiao Tong University School of Medicine, Shanghai, 200127 China

**Keywords:** Glioblastoma multiforme, Long non-coding RNA, Protein coding genes, Signature, Prognostic

## Abstract

**Background:**

Glioblastoma multiform (GBM) is a devastating brain tumor with maximum surgical resection, radiotherapy plus concomitant and adjuvant temozolomide (TMZ) as the standard treatment. Diverse clinicopathological and molecular features are major obstacles to accurate predict survival and evaluate the efficacy of chemotherapy or radiotherapy. Reliable prognostic biomarkers are urgently needed for postoperative GBM patients.

**Methods:**

The protein coding genes (PCGs) and long non-coding RNA (lncRNA) gene expression profiles of 233 GBM postoperative patients were obtained from The Cancer Genome Atlas (TCGA), TANRIC and Gene Expression Omnibus (GEO) database. We randomly divided the TCGA set into a training (*n *= 76) and a test set (*n* = 77) and used GSE7696 (*n *= 80) as an independent validation set. Survival analysis and the random survival forest algorithm were performed to screen survival associated signature.

**Results:**

Six PCGs (EIF2AK3, EPRS, GALE, GUCY2C, MTHFD2, RNF212) and five lncRNAs (CTD-2140B24.6, LINC02015, AC068888.1, CERNA1, LINC00618) were screened out by a risk score model and formed a PCG-lncRNA signature for its predictive power was strongest (AUC = 0.78 in the training dataset). The PCG-lncRNA signature could divide patients into high- risk or low-risk group with significantly different survival (median 7.47 vs. 18.27 months, log-rank test *P* < 0.001) in the training dataset. Similar result was observed in the test dataset (median 11.40 vs. 16.80 months, log-rank test *P* = 0.001) and the independent set (median 8.93 vs. 16.22 months, log-rank test *P* = 0.007). Multivariable Cox regression analysis verified that it was an independent prognostic factor for the postsurgical patients with GBM. Compared with IDH mutation status, *O*-(6)-methylguanine DNA methyltransferase promoter methylation status and age, the signature was proved to have a superior predictive power. And stratified analysis found that the signature could further separated postoperative GBM patients who received TMZ-chemoradiation into high- and low-risk groups in TCGA and GEO dataset.

**Conclusions:**

The PCG-lncRNA signature was a novel prognostic marker to predict survival and TMZ-chemoradiation response in GBM patients after surgery.

**Electronic supplementary material:**

The online version of this article (10.1186/s12967-018-1744-8) contains supplementary material, which is available to authorized users.

## Background

Glioblastoma multiforme (GBM) is regarded as the most common malignant brain tumor in adults, accounting for 47.1% of all malignant brain tumors [1], and the median survival time of untreated patients with GBM is only 3 months [2]. For malignant brain tumors, according to the Central Brain Tumor Registry of the United States (CBTRUS), the incidence rate of GBM in the United States is extremely high (3.20/100,000 population) and increases with age [1]. Maximal surgical resection, is considered as the first-line treatment for GBM patients relieving clinical symptoms, extending survival time and providing tissue to pathological diagnosis [3]. A large-scale randomized phase III trial, initiated by the European Organization for Research and Treatment of Cancer and National Cancer Institute of Canada Clinical Trials Group, found that the 2-year survival rate of GBM patients was improved to 26.5% by radiotherapy plus temozolomide from 10.4% by radiotherapy alone [4]. Since then, the standard therapeutic strategy for glioblastoma patients has become the multimodal treatment with radiotherapy and chemotherapy after surgery. Therefore, prediction of response to chemotherapy drugs or radiation and prediction of prognosis are crucial for post-surgical GBM patients.

In 1993, the Radiation Therapy Oncology Group-Recursive Partitioning Analysis (RTOG-RPA) classification system was developed for high-grade glioma patients with similar survival times [5] and validated its prognostic significance in GBM patients [6–8]. However, all the stratification variables of RTOG-RPA risk classification are clinical factors including age, tumor size and location, treatment, karnofsky performance score (KPS), cytologic, histologic composition and so on. Due to the intra- and inter-individual heterogeneity, the RTOG-RPA classification could not satisfactorily predict the survival and tumor response to therapy of each individual [9]. Therefore, molecular markers are becoming more useful in the field of prognosis prediction [10]. Currently, GBM related researches from genomics, epigenomics and transcriptomics level have led to unprecedented discoveries of potential prognostic and predictive indicators [11]. Genomic analysis suggests survival-related genomic abnormalities in GBM patients, such as epidermal growth factor receptor (EGFR) amplification [12, 13] and isocitrate dehydrogenase 1/2 (IDH1/2) mutations [14, 15], have prognostic value. Some studies show that high expression of EGFR indicated poor prognosis [16], and other research find the IDH mutations are associated with improved survival [17]. From the epigenetic level, *O*-6-methylguanine-DNA methyltransferase (MGMT) promoter methylation has been demonstrated that it is associated with improved progression-free and overall survival in GBM patients treated with alkylating agents [18]. However, genomic prognostic classification of GBM is not yet clinically feasible, and the mechanism of how these multiple genomic alterations affect clinical prognosis is not clear [19].

As far as the transcriptomics level is concerned, studies mostly focus on mRNA or protein coding gene (PCG) and long noncoding gene (lncRNA) because of their role as gene expression regulators, tumor suppressors and oncogenes. Using PCGs or lncRNAs, numerous studies have constructed transcriptome prognosis models for GBM survival prediction. Zhu et al. screened out an effective prediction system composed of 63 signature genes for glioblastoma prognosis [20]. Marko et al. identified a 43-gene “fingerprint” from a population of 1478 differential expressed genes (*P* < 0.01) that distinguished GBM survival phenotypes [21]. Anindya Dutta et al. in a global analysis identified 584 lncRNAs correlated with a poor prognosis and 282 lncRNAs associated with better survival outcomes in GBM patients [22]. Above researches verify PCGs and lncRNAs can be prognostic biomarkers of GBM. However, these studies found too many prognostic genes to provide a clinically feasible transcriptome signature with a small number of genes to predict the survival of GBM patients. Therefore, we focus our attention on find out a molecular signature which contains few prognostic genes and could more accurately predict the outcomes of postoperative GBM patients and guide the tailored therapy.

In the present study, we sought to explore the role of multi-transcriptome signature in the prognosis of GBM patients after surgery. We analyzed 233 postoperative GBM patients with the expression profiles of mRNAs and lncRNAs and screened out genes significantly associated with survival. Through further bioinformatics analysis, we aimed at constructing a prognostic transcriptome signature to divide patients into different risk groups, thereby assessing the survival and treatment response for GBM patients after surgical resection.

## Methods

### Glioblastoma multiforme datasets

We downloaded the normalized TCGA level 3 mRNA expression data and corresponding clinical information of GBM patients (*n *= 153) from the UCSC Xena (https://gdc.xenahubs.net/download/TCGA-GBM/Xena_Matrices/TCGA-GBM.htseq_fpkm.tsv.gz). LncRNA expression data of the corresponding GBM patients was obtained from the TANRIC database (https://ibl.mdanderson.org/tanric/_design/basic/download.html) [23]. Another part GBM expression data (GSE7696, *n *= 80) and corresponding clinical data was obtained from the publicly available GEO database (https://www.ncbi.nlm.nih.gov/geo/). GSE7696 data was generated by the Affymetrix Human Genome U133 Plus 2.0 Array (http://www.affymetrix.com/support/technical/byproduct.affx?product=hg-u133-plus) and included 80 tumors and 4 normal samples. By probe re-annotation [24], we got their PCG and lncRNA expression data. Then we processed the gene expression data by removing the genes with missing expression values in more than 30% of samples or patients and excluding genes whose expression value were 0 or null [25]. For the remaining genes with missing expression value in less than 30% of samples or patients, we used the mean of the corresponding genes expression values by R program to replace the missing expression values. We used the expression value on a log2 scale in the subsequent analysis.

A total of 233 glioblastoma patients concurrent with gene expression profiles and clinical information were utilized in our study. Of these, all GBM patients were postoperative, then treated with radiotherapy or chemotherapy. The 153 GBM patients from TCGA database were randomly assigned to a training set (*n* = 76) or a testing set (*n *= 77) using the ‘sample’ function [26] from R library and the 80 patients from GSE7696 were served as an independent validation set. Table 1 described the clinical characteristics and therapy information of the TCGA and GEO cohort respectively.

**Table 1:** Summary of patient demographics and clinical characteristics

Characteristic	Training set	Test set	Independent set
Age			
≤ 60	41	35	64
> 60	35	42	16
Sex			
Female	26	29	21
Male	50	48	59
Chemotherapy			
No	14	15	28
Yes	53	55	52
Unknown	9	7	0
Radiotherapy			
No	8	13	0
Yes	62	63	80
Unknown	6	1	0
TMZ-chemoradiation			
No	41	42	28
Yes	33	29	52
Unknown	2	6	0
Subtype			
Classical	18	22	
Mesenchymal	23	26	
Neural	15	11	
Proneural	20	17	

### Construction of the prognostic PCG-lncRNA signature in the training dataset

The relationship between the expression of PCG or lncRNA and patients’ overall survival (OS) was analyzed by univariate Cox proportional hazards regression analysis in the training dataset. Genes were selected if P value < 0.05. Before constructing a risk prediction model, the random survival forests-variable hunting (RSFVH) algorithm was performed to filter genes. In the random survival forest supervised classification algorithm, an iteration procedure was implemented to narrow down the gene set, and each iteration step discarded the 1/4 least important PCGs or lncRNAs. One thousand trees were grown at each step, and the square root of the number of input nodes at each step was set to the size of randomly chosen PCGs or lncRNAs at each node of single classification tree. Because the number of good-prognostic and poor-prognostic patients were not equal, the class weights were adjusted accordingly. The generalization error was estimated on the out-of-bag samples. Finally, six PCGs and six lncRNAs were selected [27–29]. Risk prediction score model was developed by these selected genes, weighted by their estimated regression coefficients as follows [30].

$${\text{Risk}}\;{\text{Score}}\;\left( {{\text{RS}}} \right) = \sum\limits_{{{\text{i}} = 1}}^{{\text{N}}} {\left( {Exp_{{\text{i}}} \;*\;Co{\text{e}}_{{\text{i}}} } \right)}$$

where N is the number of prognostic lncRNAs or PCGs, *Ex*p_i_ is the expression value of lncRNAs or PCGs, and *Co*e_i_ is the estimated regression coefficient of PCGs or lncRNAs in the univariate Cox regression analysis. Then each patient obtained 4095 risk scores because six PCGs and six lncRNAs could form 2^12^ − 1 = 4095 combinations or signatures. The receiver operating characteristic (ROC) curve was used to compare the sensitivity and specificity of the 4095 signatures in the training dataset. Area under the curve (AUC) were calculated from the ROC curve. By comparing the AUC values, we selected the prognostic PCG-lncRNA signature in the training set.

### Statistical analysis and bioinformatics analysis

With the median risk score in the training dataset as the cutoff value, the GBM patients in training or test set were divided into high-risk or low-risk group [31]. In GSE7696, X-tile software was used to select cutoff value for risk grouping [32]. The Kaplan–Meier analysis and the log-rank test were used to assess and compare survival differences between the low-risk and high-risk groups. ROC analysis was tested to compare the survival predictive power. Furthermore, to test whether the signature was an independent prognostic factor, multivariable Cox regression analysis and data stratification analysis were performed. All analyses were performed using R program 3.2.3 (http://www.r-project.org) including packages named pROC, survival and randomForestSRC downloaded from Bioconductor.

To investigate the biological roles of the PCGs and lncRNAs in the signature, we analyzed the co-expressed protein coding genes of the prognostic genes computed by Pearson correlation test and genes with P < 0.05 and absolute value of the Pearson coefficient > 0.4 were selected. Here, SubpathwayMiner was used for identification of related pathways of the selected genes (http://cran.r-project.org/web/packages/SubpathwayMiner/) for it supports multiple species (approximately 100 eukaryotes, 714 bacteria and 52 Archaea) and different gene identifiers (Entrez Gene IDs, NCBI-gi IDs, UniProt IDs, PDB IDs, etc.) in the KEGG GENE database, which provides more flexibility in annotating gene sets and identifying the involved pathways (entire pathways and sub-pathways) [33].

## Results

### Characteristics of study subjects

In this study, the GBM patients after surgical resection and their expression profiles were used as the main subjects. After screened the data downloaded from the TCGA, TANRIC and GEO database, we identified 233 eligible patients diagnosed with GBM concurrently including PCG and lncRNA expression profiles and corresponding clinical data. All these GBM patients received surgical treatment and the median age of the enrolled patients was 60 years (21–89 years). Simultaneously, we obtained a total of 14,607 PCGs and 6613 lncRNAs from the 233 GBM patients.

### Identification of the prognostic PCG-lncRNA signature in the training dataset

**Fig. 1:**
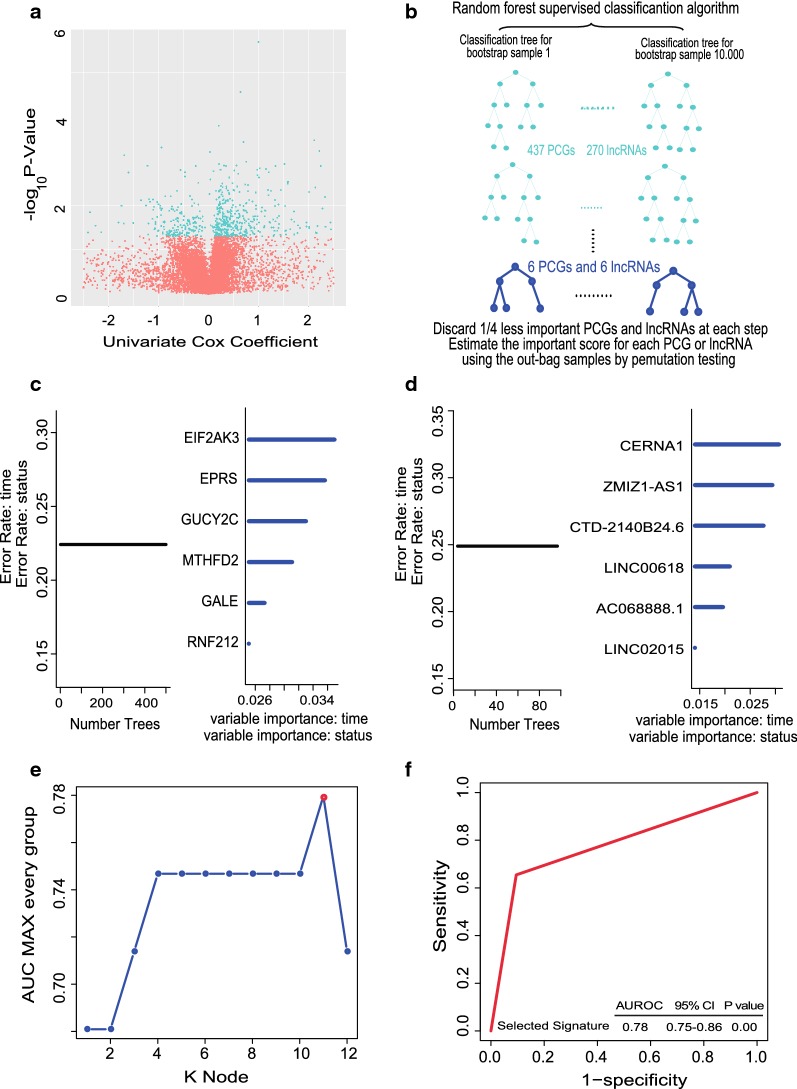
Identification of the prognostic PCG-lncRNA signature in the training dataset. **a** Volcano plot of the survival associated lncRNAs and PCGs in univariate cox regression analysis. **b** The random survival forest analysis of the survival related 707-gene set in postsurgical GBM patients. **c**, **d** The important score of selected PCGs or lncRNAs which were calculated by random survival forest analysis. **e** The 11 genes with largest AUC (k = 1, 2, … 12, k represents the gene number in the corresponding signature) were shown in the plot. **f** After calculating the AUC of 4095 signatures, the prognostic PCG-lncRNA signature with biggest predictive power (AUC = 0.78) was screen out

Firstly, in order to find the survival-related genes in training set, univariate cox proportional hazards regression analysis was performed and identified a 707-genes set including 437 PCGs and 270 lncRNAs in the training dataset which were significantly correlated with OS (*P* < 0.05, Additional file 1: Table S1). The volcano plot displayed the 707 genes with statistical differences as the blue dots in Fig. 1a. Secondly, to further narrow down the number of prognostic PCGs or lncRNAs, we analyzed the above 707 survival related genes by random survival forest algorithm and got six PCGs (EIF2AK3, EPRS, GALE, GUCY2C, MTHFD2, RNF212) and six lncRNAs (LINC00618, LINC02015, AC068888.1, CERNA1, CTD-2140B24.6, ZMIZ1-AS1) significantly associated to OS of GBM patients according to the permutation important score in every step: Discard 1/4 less important PCGs and lncRNAs at each step based on estimating the important score for each PCG or lncRNA using the out-bag samples by pemutation testing (Fig. 1b–d).

Thirdly, putting the six lncRNAs and six PCGs into the risk score model constructed in methods, we obtained a total of 2^12^ − 1 = 4095 models or signatures that included different gene numbers from 1 to 12, indicating that PCGs and lncRNAs alone or the combination of PCGs and lncRNAs were included in these 4095 models. To screen out a signature with biggest predictive power, we performed ROC analysis 4095 times using the survival status as label and signature risk scores of GBM patients as variable in the training dataset by pROC R packages. After compared the AUC values of all these 4095 signatures (Additional file 2: Table S2), we identified the max AUC value was 0.78 (Fig. 1e, f) from the PCG-lncRNA signature comprising six PCGs (EIF2AK3, EPRS, GALE, GUCY2C, MTHFD2, RNF212) and five lncRNAs (LINC00618, LINC02015, AC068888.1, CERNA1 and CTD-2140B24.6).

The risk score model was constructed as follows: Risk score = (− 0.82 × EIF2AK3 expression) + (− 0.79 × EPRS expression) + (0.71 × GALE expression) + (0.60 × GUCY2C expression) + (− 0.71 × MTHFD2 expression) + (− 0.87 × RNF212 expression) + (0.57 × LINC00618 expression) + (− 0.84 × LINC02015 expression) + (0.61 × AC068888.1 expression) + (0.65 × CERNA1 expression) + (− 0.74 × CTD-2140B24.6 expression). Among them, the coefficients for PCGs (EIF2AK3, EPRS, MTHFD2 and RNF212) and lncRNAs (LINC02015, CTD-2140B24.6) are negative, and the coefficients for PCGs (GALE, and GUCY2C) and lncRNAs (LINC00618, AC068888.1 and CERNA1) are positive (Table 2).

**Table 2:** Identities of PCGs and lncRNAs in the prognostic PCG-lncRNA signature and their univariable cox association with prognosis in the training group

Ensembl database ID	Gene symbol	Coefficient ^a^	*P* value ^a^	Gene expression level association with poor prognosis
ENSG00000172071	EIF2AK3	− 0.82	< 0.001	Low
ENSG00000136628	EPRS	− 0.79	0.01	Low
ENSG00000065911	MTHFD2	− 0.71	0.02	Low
ENSG00000178222	RNF212	− 0.87	0.00	Low
ENSG00000117308	GALE	0.71	0.01	High
ENSG00000070019	GUCY2C	0.6	0.03	High
ENSG00000231574	LINC02015	− 0.84	< 0.001	Low
ENSG00000271963	CTD-2140B24.6	− 0.74	0.01	Low
ENSG00000225163	LINC00618	0.57	0.04	High
ENSG00000257337	AC068888.1	0.61	0.03	High
ENSG00000259577	CERNA1	0.65	0.02	High

^a^Derived from the univariable Cox regression analysis in the training group

### Validation the survival prediction of the PCG-lncRNA signature in the three dataset

**Fig. 2:**
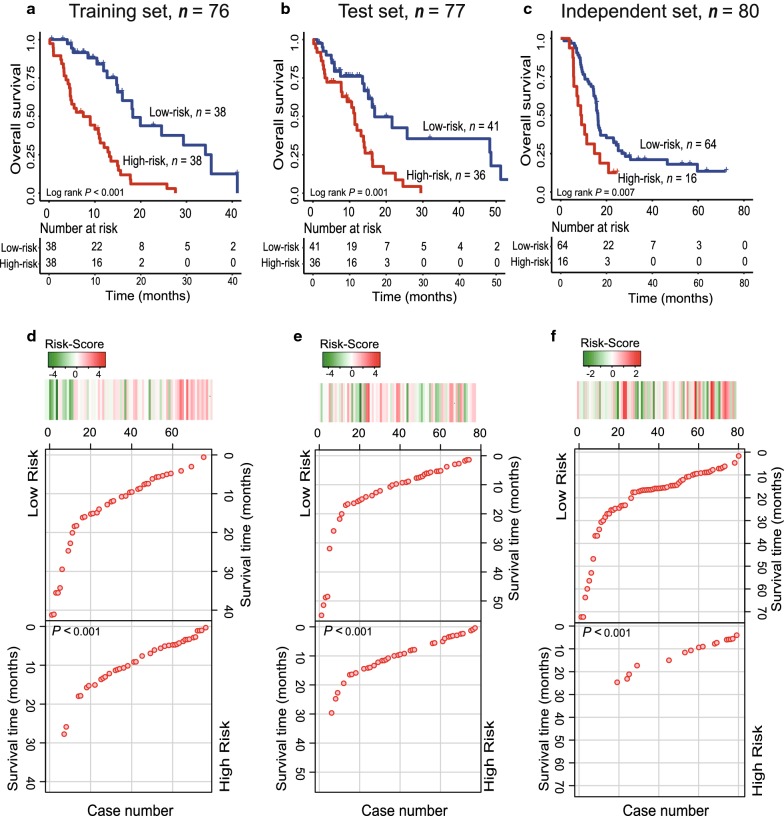
The PCG-lncRNA signature predicts survival of postoperative GBM patients in the training, test and independent validation set. Kaplan–Meier survival curves classify training-set patients (*n *= 76) (**a**) test-set patients (*n *= 77) (**b**) and independent validation set (*n *= 80) (**c**) into high- and low-risk groups by the PCG-lncRNA signature. Shorter survival time was noted in GBM patients with higher risk-scores in the training (**d**), test (**e**) and independent validation (**f**) datasets

The risk score model constructed by the PCG-lncRNA signature in the training dataset gave each patient a risk score. Patients from the training dataset were divided into high-risk group (*n* = 38) and low-risk group (*n* = 38) when the median risk score was used as the cutoff point. Kaplan–Meier survival analysis was performed to compare the overall survival of two risk groups of patients. As we can see in Fig. 2a, the OS rates were significantly different in patients from the two groups. Compared with those in the low-risk group, patients in the high-risk group had a shorter survival time (median survival: 7.47 months vs. 18.27 months, log-rank test *P *< 0.001) and lower OS rate (5% vs. 50%, log-rank test *P *< 0.001, Fig. 2a).

To validate the predictive power of the signature, we calculated the PCG-lncRNA signature-based risk scores of 77 patients in the test dataset. When the same median cutoff point obtained from the training dataset was used, patients from the test dataset were also separated into low-risk and high-risk groups (median survival: 11.4 months vs. 16.8 months, log-rank test *P* = 0.001, Fig. 2b). The OS rate of patients in the high-risk group was about 19.4% vs. 53.7% in the low-risk group (Fig. 2b). In the independent set, Kaplan–Meier analysis found the PCG-lncRNA signature classified patients into different two risk groups (median survival: 8.93 months vs. 16.22 months, log-rank test *P* = 0.007, Fig. 2c). Moreover, shorter survival time was noted in GBM patients with higher risk-scores in the training, test and independent datasets and *P*-values were calculated by the rank-sum test (Fig. 2d–f).

### The PCG-lncRNA signature is an independent prognostic factor from other clinical variables and molecular features

After demonstrating the survival predictive power of PCG-lncRNA signature, we need to clarify whether the PCG-lncRNA signature was an independent prognostic factor since numerous factors affect GBM prognosis. Thus we performed univariable cox analysis and multivariable Cox regression analysis in which covariates included the PCG-lncRNA signature-based risk score and clinical features (Table 3). Multivariable Cox regression analysis showed that the PCG-lncRNA risk score remained to be significantly associated with overall survival when adjusted other clinical features including sex, age and Karnofsky performance score in the training and the test dataset (High-risk group vs. Low-risk group, HR = 5.94, 95% CI 2.66–13.25, *P *< 0.001; HR = 2.89, 95% CI 1.35–6.20, *P *= 0.01). The independent dataset showed the independent predictive power of PCG-lncRNA signature (HR = 2.17, 95% CI 1.16–4.07, P = 0.02) when adjusted other clinical features including sex, age.

**Table 3:** Univariable and multivariable Cox regression analysis of the PCG-lncRNA signature and survival of GBM patients

Variables	Univariable analysis				Multivariable analysis			
	HR	95% CI of HR		*P*	HR	95% CI of HR		*P*
		Lower	Upper			Lower	Upper	
The training set								
Age								
> 60 vs. ≤ 60	1.54	0.9	2.64	0.12	1.96	0.98	3.92	0.06
Sex								
Male vs. female	0.68	0.39	1.2	0.18	0.65	0.33	1.28	0.21
KPS								
> 70 vs. ≤ 70	0.86	0.42	1.74	0.04	2.48	1.05	5.87	0.04
The signature								
High risk vs. low risk	4.68	2.48	8.80	< 0.001	5.94	2.66	13.25	< 0.001
The test set								
Age								
> 60 vs. ≤ 60	1.93	1.05	3.56	0.04	1.66	0.81	3.44	0.16
Sex								
Male vs. female	0.8	0.45	1.43	0.45	1.16	0.52	2.59	0.71
KPS								
> 70 vs. ≤ 70	0.51	0.25	1.07	0.08	0.53	0.24	1.17	0.12
The signature								
High risk vs. low risk	2.77	1.49	5.16	0.001	2.89	1.35	6.20	0.01
The independent set								
Age								
> 60 vs. ≤ 60	1.65	0.92	2.96	0.09	1.54	0.85	2.79	0.15
Sex								
Male vs. female	0.92	0.53	1.61	0.77	1.05	0.59	1.85	0.88
The signature								
High risk vs. low risk	2.24	1.22	4.11	0.01	2.17	1.16	4.07	0.02
The entire TCGA set								
MGMT								
Methylated vs. unmethylated	0.92	0.48	1.77	0.80	0.94	0.49	1.83	0.87
IDH1								
R132H vs. WT	0.27	0.04	2.01	0.20	0.46	0.06	3.57	0.46
The signature								
High risk vs. low risk	4.00	1.97	8.12	< 0.001	3.71	1.80	7.62	< 0.001

*KPS* Karnofsky performance score

Subsequently, examining the clinical data of these 153 TCGA GBM patients after surgical resection, we obtained 73 samples with known status of MGMT promoter and 80 samples with known status of IDH1 mutation (both known were 70 samples). Multivariable Cox regression analysis showed that the PCG-lncRNA risk score was significantly associated with overall survival when adjusted the molecular features including MGMT promoter and IDH1 mutation in the 70 TCGA GBM patients (High-risk group vs. Low-risk group, HR = 3.71, 95% CI 1.80–7.62, *P *< 0.001).

### Comparing the survival predictive power of the signature with that of age, IDH1 mutation and MGMT promoter methylation status

To compare the survival predictive power of the PCG-lncRNA signature with the reported prognostic factors, such as age, IDH1 mutation and MGMT promoter methylation status, we performed a series of ROC analyses considering that a larger AUC usually represented a better predictive power [34, 35].

**Fig. 3:**
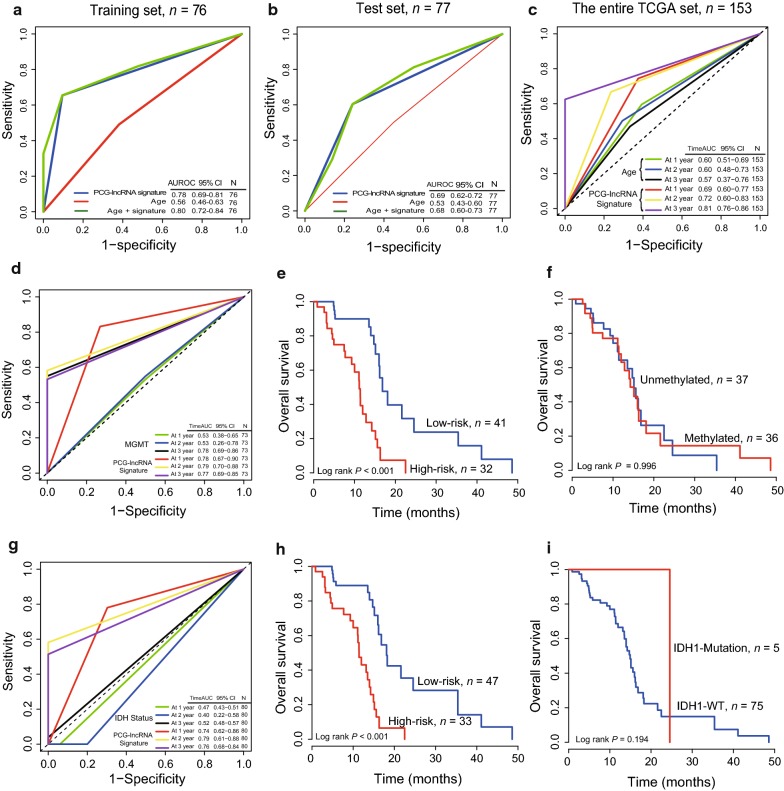
ROC analyses for comparison of the predictive power of the PCG-lncRNA signature with that of age in the training (**a**), test dataset (**b**) and TimeROC analysis in the entire set (**c**). TimeROC analysis for comparison of the survival predictive power of the PCG-lncRNA signature with that of MGMT promoter methylation status (**d**) and IDH1 mutation (**g**). Kaplan–Meier survival curves found the PCG-lncRNA signature (**e**, **h**) could classify patients with known MGMT promoter (**f**) and IDH1 mutation status (**i**) into high- and low-risk groups by the PCG-lncRNA signature in each corresponding TCGA dataset. Kaplan–Meier survival curves found the MGMT and IDH1/2 could not group patients into high- and low-risk groups

In the training dataset (*n* = 76), the AUC of the PCG-lncRNA signature was bigger than that of age, indicating a better predictive power in GBM prognosis (Signature-AUC = 0.78 vs. Age-AUC = 0.56, Fig. 3a). The same result can be seen in the test dataset (Signature-AUC = 0.69 vs. Age-AUC = 0.53, *n *= 77, Fig. 3b), Furthermore, the AUC of the signature model combined with age was maximum (Age + Signature-AUC = 0.80/0.68 in training/test group, Fig. 3a, b), illustrating combination of the PCG-lncRNA signature with age could provide more precisely prognostic information. And we also compared the survival predictive ability at 1, 2 and 3 years of the PCG-lncRNA signature with that of age by TimeROC analysis in the entire TCGA 153 samples. As Fig. 3c showed, the AUC of the PCG-lncRNA signature is 0.69 (0.60–0.77) at 1 year, 0.72 (0.60–0.83) at 2 year and 0.81 (0.76–0.86) at 3 year, larger than that of age 0.60 (0.51–0.69) at 1 year, 0.60 (0.48–0.73) at 2 year and 0.57 (0.37–0.76) at 3 year.

Then, we compared the survival predictive power of the PCG-lncRNA signature with MGMT or IDH1 mutation by TimeROC analysis. For MGMT set (*n *= 73), the AUC of the signature was 0.78 (0.67–0.90) at 1 year and 0.79 (0.70–0.88) at 2 year, larger than that of MGMT which was 0.53 (0.38–0.65) at 1 year and 0.53 (0.26–0.78) at 2 year, but the AUC of the signature was 0.77 (0.69–0.85) at 3 year a little less than that of MGMT which was 0.78 (0.69–0.86) (Fig. 3d). For IDH1 mutation set (*n *= 80), the AUC of the PCG-lncRNA signature was 0.74 (0.62–0.86) at 1 year, 0.79 (0.61–0.88) at 2 year and 0.76 (0.68–0.84) at 3 year, larger than that of IDH1 mutation which was 0.47 (0.43–0.51) at 1 year, 0.40 (0.22–0.58) at 2 year and 0.52 (0.48–0.57) at 3 year (Fig. 3g).

In addition, Kaplan–Meier survival analysis was performed in the 73 samples with known status of MGMT promoter and 80 samples with known status of IDH1 mutation to compare the risk grouping ability of the PCG-lncRNA signature with that of MGMT and IDH1 mutation. Using the same median cutoff point obtained from the training dataset, the PCG-lncRNA signature showed a robust efficiency to separate corresponding patients into two risk groups with different survival time (*P *< 0.001, Fig. 3e, h), however, the MGMT and IDH1/2 did not group well (*P *> 0.05, Fig. 3f, i).

### Stratification analysis of TMZ-chemoradiation treatment

**Fig. 4:**
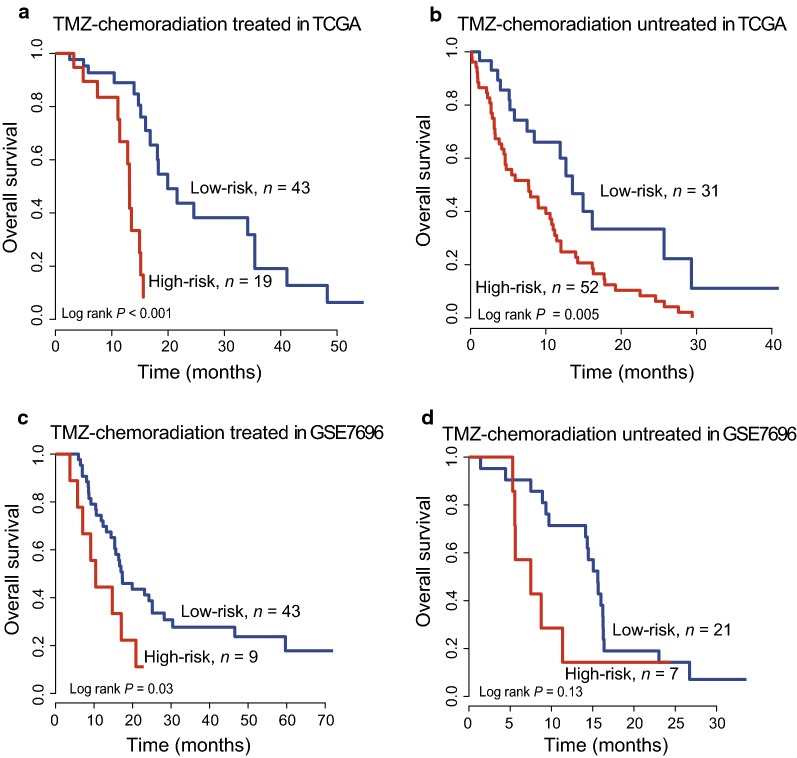
Stratification analysis in the entire set. Kaplan–Meier survival curves found the PCG-lncRNA signature could classify TMZ-chemoradiation treated patients (**a**) and untreated patients (**b**) in the entire TCGA dataset and TMZ-chemoradiation treated patients (**c**) and untreated patients (**d**) in the GSE7696 dataset into high- and low-risk groups. P Value was calculated by log-rank test

The relationship between the PCG-lncRNA signature with a series of clinicopathological parameters in the entire TCGA dataset (*n *= 153) was analyzed. As can be seen in Table 4, there was an association between PCG-lncRNA signature and TMZ-chemoradiation (Chi square test, *P* < 0.05, Table 4). Obviously, TMZ-chemoradiation treatment could stratify post-operative GBM patients into treated stratum and untreated stratum. Data stratification analysis using the PCG-lncRNA signature risk score further divided the patients into four groups: high-risk and treated, high-risk and untreated, low-risk and treated, low-risk and untreated. The Kaplan–Meier test was performed and Kaplan–Meier curves showed in Fig. 4. The log-rank test showed that TMZ-chemoradiation treated patients in high-risk group with shorter survival than TMZ-chemoradiation treated patients in low-risk group (*n *= 62, *P *< 0.001, Fig. 4a). The TMZ-chemoradiation untreated patients were also divided into a high-risk group with lower OS and a low-risk group with higher OS (*n *= 83, *P* = 0.005, Fig. 4b), indicating the stratification power of the PCG-lncRNA signature in TMZ-chemoradiation GBM patients.

**Table 4:** Association of the PCG-lncRNA signature with clinicopathological characteristics in postoperative GBM patients in TCGA dataset

Variables	Training set		*P*	Test set		*P*	Entire TCGA set		*P*
	Low risk^a^	High risk^a^		Low risk^a^	High risk^a^		Low risk^a^	High risk^a^	
Age			0.76			1			0.93
< 60	21	20		19	16		40	36	
≥ 60	17	18		22	20		39	38	
Sex			0.23			0.97			0.52
Female	10	16		16	13		26	29	
Male	28	22		25	23		53	45	
TMZ-chemotherapy			0.16			0.51			0.11
No	4	10		6	9		10	19	
Yes	30	23		31	24		61	47	
Unknown	4	5		4	3		8	8	
Radiotherapy			0.01			0.26			0.01
No	1	7		5	8		6	15	
Yes	36	26		36	27		72	53	
Unknown	1	5		0	1		1	6	
TMZ-chemoradiation			0.01			0.03			< 0.001
No	14	27		17	25		31	52	
Yes	23	10		20	9		43	19	
Unknown	1	1		2	4		3	5	
Subtype			0.8			0.34			0.47
Classical	9	9		8	14		17	23	
Mesenchymal	13	10		15	11		28	21	
Neural	6	9		6	5		12	14	
Proneural	10	10		11	6		21	16	

^a^Low risk ≤ median value of the PCG-lncRNA signature risk score, high risk > median of risk score in training group; The Chi-squared test; *P* value < 0.05 was considered significant; *TMZ* temozolomide

In consistence with the findings in TCGA described above, for GSE7686 dataset, the PCG-lncRNA signature could stratify the TMZ-chemoradiation treated or untreated patients into a high-risk group and a low-risk group with different survival (log-rank test *P *= 0.03, Fig. 4c, log-rank test *P *= 0.13, Fig. 4d).

### Functional characterization of the prognostic genes in the PCG-lncRNA signature

**Fig. 5:**
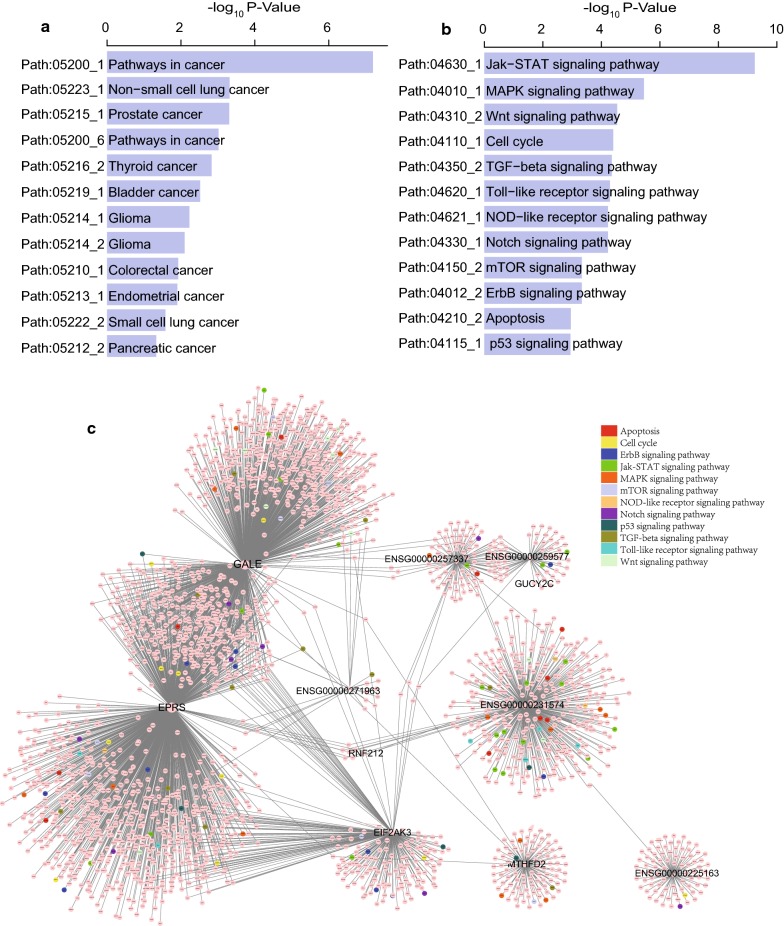
Pathway analysis of the co-expressed protein-coding genes in the PCG-lncRNA signature. **a**, **b** Significantly enriched pathways. **c** The co-expression network

Co-expression network analysis was carried out in the entire TCGA dataset visualized by Cytoscape [36] and we found 2328 protein-coding genes co-expressed with the prognostic 6 PCGs and 5 lncRNAs in the signature (Absolute value of the Pearson correlation coefficient > 0.40, *P* < 0.05, Additional file 3: Table S3). Then we performed pathway analysis by SubpathwayMiner (see method) and found these co-expressed genes were enriched in 90 different pathways (*P* < 0.05, Additional file 4: Table S4). The gene set were significantly associated with different cancer types such as non-small cell lung cancer, prostate cancer, thyroid cancer, bladder cancer and glioma (*P *< 0.05, Fig. 5a). And these results suggested that the 11 genes, via the co-expressed genes, could exert their regulatory roles by implicating in regulating downstream pathways such as JAK-STAT signaling pathway, MAPK signaling pathway, WNT signaling pathway, Cell cycle, TGF-beta signaling pathway and p53 signaling pathway (*P* < 0.05, Fig. 5b, c).

## Discussion

Glioblastoma multiforme (GBM) is a heterogeneous disease characterized by poor prognosis. In order to extend the survival time of patients with GBM, in recent year, adjuvant and concomitant temozolomide with radiation are widely used. Despite advances in treatment such as radiation and chemotherapy, the prognosis and therapy response for post-surgical GBM patients with similar clinical risk factors varied tremendously. Considering the molecular heterogeneity of GBM, in this study, we identified a prognostic molecular indicator comprising five long non-coding RNAs and six protein coding genes, and confirmed the survival prediction power of the PCG-lncRNA signature in postoperative GBM patients.

Molecular markers are of great significance to disease diagnosis, treatment decision and prognosis assessment. With regard to the prognostic molecular characterization of GBM, the 2016 World Health Organization (WHO), for the first time, used the isocitrate dehydrogenase (IDH) gene mutation status as the classification molecular parameter to separate the GBM into three groups: GBM IDH-wild type, GBM IDH-mutant, and GBM NOS [37], with different prognosis [38]. In the past decade, GBM prognostic studies focused on mRNA or PCG as a result of the development of sequence technology and The Cancer Genome Atlas (TCGA) database. Chen et al. selected a gene expression signature score (GGESS) by incorporating ten glycolytic genes significantly correlated with patient survival and verified that the PCG signature could independently predict prognosis and response to chemotherapy of GBM patients [39]. According to Chinese Glioma Genome Atlas (CGGA) RNA sequencing database and TCGA DNA methylation, another study established a gene signature comprising eight differentially expressed genes affected by DNA methylation and validated its prognostic value for GBM patients [40]. A minimal multigene signature that correlated with patient survival and effectively separated the proneural and mesenchymal glioblastoma subtypes was developed from two patient-derived novel primary cell culture models (MTA10 and KW10) [41].

Recently, emerging evidence suggests that lncRNA play a vital role in cancer occurrence and development, such as regulating gene transcription [42] and post transcriptional processing of mRNA [43], participating in chromatin remodeling [44]. Subsequently, a great deal of lncRNAs have been shown to be closely associated with the survival of patients in different cancer types, indicating its prognostic prediction role. For GBM patients, some researchers identified a six-lncRNA signature associated with the overall survival by analyzing lncRNA expression profiling in 213 GBM tumors from TCGA [45]. An immune-related six-lncRNA signature was found by performing a genome-wide analysis of lncRNA expression profiles form 419 GBM patients and demonstrated its ability to stratify patients into high- and low-risk groups with significantly different survival [46]. All these above mentioned studies highlighted that it is feasible to mine the reliable and readily available expression profiles from TCGA database in GBM prognostic PCG/lncRNA marker studies. Moreover, a recent work found the dysregulated lncRNAs and mRNAs associated with acquired TMZ resistance in glioblastoma cells in vitro and may provide novel targets for GBM chemotherapy [47].

Therefore, in the present study, we combined the PCG expression profile with the tissue-specific lncRNA expression profile to explore a signature indicating the prognosis and therapy effectiveness of postoperative GBM patients. We obtained 233 postsurgical GBM patients with corresponding PCG, lncRNA expression profiles and clinical information as the study object. After summarized clinical characteristics, we found the median age of the postsurgical GBM patients was 60 and more common in men, almost consistent with most research reported [48–50]. Clinical treatment information of these 233 GBM patients provided convenience for our research on treatment response. Subsequently, we used two powerful bioinformatics analysis methods for identification of prognostic genes. Firstly, univariable cox regression analysis was performed and identified 707 genes that was significantly associated with the overall survival of GBM patients in the training dataset. Secondly, the random survival forest method further minimized the prognostic genes to 6 PCGs and 6 lncRNAs. Then we screened out a PCG-lncRNA signature with biggest AUC from 4095 combinations including different number of PCGs and/or lncRNAs, comprising six PCGs (EIF2AK3, EPRS, GALE, GUCY2C, MTHFD2, RNF212) and five lncRNAs (LINC00618, LINC02015, AC068888.1, CERNA1, CTD-2140B24.6), which separated patients into low-risk or high-risk group with different survival in the training and test dataset. The biggest AUC value of the PCG-lncRNA signature suggests it was better than any PCG alone signature or lncRNA alone signature. Multivariable Cox regression analysis verified the independence of the selected PCG-lncRNA signature from clinical factors like sex, age, KPS in predicting survival in postoperative GBM patients. As we mentioned, radiotherapy plus concomitant and maintenance TMZ chemotherapy after operation is the standard treatment for GBM patients, which means most postoperative GBM patients experienced TMZ-chemoradiation. Notably, the stratification analysis found that the PCG-lncRNA signature could further classify the TMZ-chemoradiation patients into low-risk or high-risk group with different survival, indicating the PCG-lncRNA signature could be helpful in predicting GBM treatment outcome, especially in TMZ-chemoradiation treated patients. Previous studies reported that age, MGMT promoter and IDH1 mutation were one of the main prognostic factors for GBM [45], so we compared the predictive ability of age, MGMT promoter and IDH1 mutation with that of the PCG-lncRNA signature, and the ROC analysis results confirmed the signature had a superior survival predictive power.

To further explore the characteristics of the prognostic PCGs and lncRNAs in the signature, we found EIF2AK3, EPRS, MTHFD2, RNF212, LINC02015, CTD-2140B24.6 were protected factors for GBM patients highly expressed these genes with a long survival time (univariable cox coefficient < 0), and the remaining genes (GALE, GUCY2C, LINC00618, AC068888.1, CERNA1) associated with short survival time were risk factors (univariable cox coefficient > 0) according to the univariable cox result in Table 2. Due to relevant functional research of the prognostic 11 genes are limited, we performed bioinformatics functional analyses including co-expression network analysis and pathway analysis. However, the biological roles of the selected genes in tumorigenesis are still not clear and should be investigated in further experimental studies.

There are some limitations in this work. Firstly, after rejecting missing data, only 6613 lncRNAs were included, which might neglect some potential lncRNAs. Secondly, only 233 patients were included in the analysis, thus the efficiency of the PCG-lncRNA signature should be confirmed in more GBM patients. Moreover, the molecular mechanisms how these prognostic genes or the PCG-lncRNA signature influence patients risk stratification and clinical treatment responses need to be explained.

Although the above shortcomings, this article still has advantages and novelty. Firstly, we used few genes which predict survival and construct a PCG-lncRNA signature with satisfactorily prognosis predictive power, giving the postoperative GBM patients and clinicians a potential signature to evaluate survival. Secondly, in the post-operative GBM patients, treated with radiotherapy or chemotherapy, we found the stratification power of the signature in TMZ-chemoradiation, which is helpful for clinical treatment guiding.

## Conclusion

This is, to our knowledge, the first study investigating a correlation between the PCG-lncRNA signature and the survival in postoperative GBM patients. Our study strongly suggests that the PCG-lncRNA signature could serve as novel biomarkers for predicting prognosis and treatment outcome of postoperative GBM patients.

## Additional files

**Additional file 1: Table S1.** PCGs and lncRNAs of Univariate Cox regression analysis (P < 0.05) in the training set (n = 76).

**Additional file 2: Table S2.** The 4095 signatures composed of different PCGs and lncRNAs in the training and test dataset.

**Additional file 3: Table S3.** 2328 genes co-expressed with the 11 molecular markers in the PCG-lncRNA signature (Pearson correlation coefficient > 0.4, P < 0.05, Entire group, n = 153).

**Additional file 4: Table S4.** Pathway analysis of genes co-expressed with the prognostic PCGs or lncRNA in the signature by SubpathwayMiner.

## Abbreviations

ROC: receiver operating characteristic
AUC: area under the ROC curve
PCG: protein coding gene
LncRNA: long non-coding RNA
OS: overall survival
GBM: glioblastoma multiforme
SD: standard deviation
TCGA: The Cancer Genome Atlas
